# Influence of Biodentine^®^ - A Dentine Substitute - On Collagen Type I Synthesis in Pulp Fibroblasts *In Vitro*

**DOI:** 10.1371/journal.pone.0167633

**Published:** 2016-12-09

**Authors:** Frangis Nikfarjam, Kim Beyer, Anke König, Matthias Hofmann, Manuel Butting, Eva Valesky, Stefan Kippenberger, Roland Kaufmann, Detlef Heidemann, August Bernd, Nadja Nicole Zöller

**Affiliations:** 1 Department of Dermatology, Venereology and Allergology, Johann Wolfgang Goethe-University, Frankfurt/Main, Germany; 2 Department of Operative Dentistry, Center for Dentistry and Oral Medicine (Carolinum), Johann Wolfgang Goethe-University, Frankfurt/Main, Germany; Oklahoma State University Center for Health Sciences, UNITED STATES

## Abstract

Preserving a patient’s own teeth—even in a difficult situation—is nowadays preferable to surgical intervention and therefore promotes development of suitable dental repair materials. Biodentine^®^, a mineral trioxide aggregate substitute, has been used to replace dentine in a bioactive and biocompatible manner in both the dental crown and the root. The aim of our study was to evaluate the influence of Biodentine^®^ on pulp fibroblasts *in vitro*. For this study, one to five Biodentine^®^ discs with a diameter of 5.1mm were incubated in DMEM. To obtain Biodentine^®^ suspensions the media were collected and replaced with fresh medium every 24h for 4 days. Primary pulp cells were isolated from freshly extracted wisdom teeth of 20–23 year old patients and incubated with the Biodentine^®^ suspensions. Proliferation, cell morphology, cell integrity and cell viability were monitored. To evaluate the effect of Biodentine^®^ on collagen type I synthesis, the secretion of the N-terminal domain of pro-collagen type I (P1NP) and the release of transforming growth factor-β1 (TGF-β1) were quantified. None of the Biodentine^®^ suspensions tested influenced cell morphology, proliferation or cell integrity. The cell viability varied slightly depending on the suspension used. However, the concentrations of P1NP of all pulp fibroblast cultures treated for 24h with the moderate to high Biodentine^®^ concentration containing suspensions of day 1 were reduced to 5% of the control. Furthermore, a significant TGF-β1 reduction was observed after treatment with these suspensions. It could be shown that Biodentine^®^ is biocompatible. However, dissolved particles of the moderate to high concentrated Biodentine^®^ suspensions 24h after mixing induce a significant reduction of TGF-β1 release and reduce the secretion of collagen type I of primary pulp fibroblasts.

## Introduction

Modern dental research increasingly considers teeth and oral health within the context of the whole organism [1–4] and this approach also applies to dental conservation. In the case of teeth which would have been extracted in recent years, today even time- and resource-consuming procedures are performed to conserve the original tooth rather than replacing it with pontics and implants. Therefore tissue repair, regeneration and replacement are becoming progressively more important. In this context, pulp injury presents a particular challenge. As a specific biocompatible and bioactive material for pulp injury treatment, calcium hydroxide preparations and Mineral Trioxide Aggregate (MTA) are used. Both stimulate the development of a hard-tissue seal [5]. Since 2011, Biodentine^®^, a new bioactive repair and dentine-substitute containing similar components to MTA [6–8] has been available. Biodentine^®^ is a cement-like dental material with a wide range of applications e.g. to replace the dental dentine in any part of a dental crown or root [9–11]. It consists mainly of tricalcium silicate, dicalcium silicate and calcium carbonate [12] dissolved in calcium chloride and polycarboxylate-containing water. Due to its phosphate cement-like consistency, exchange of ions is possible [13]. Dentine etching [14] is not necessary before application. Biodentine^®^ is in direct contact with pulp fibroblasts and therefore it is of great interest to investigate the impact of Biodentine^®^ and its components on human pulp fibroblasts, in particular, the effect on biocompatibility and bioactivity. Collagen type I is a main component of the pulpal extracellular matrix. It accelerates mineralization and regulates gene expression [15] as does TGF-β1 [16, 17].

Aim of this study was to investigate the impact of Biodentine^®^ on the physiology of isolated primary pulp fibroblasts. We could show that cell viability as well as collagen type I synthesis and TGF-β1 secretion were concentration dependently regulated.

## Materials and Methods

### Cell isolation

Primary pulp fibroblasts were isolated from freshly extracted wisdom teeth of 20–23 year-old patients. Wisdom teeth were transferred directly after extraction to PBS with Ca^2+^, Mg^2+^ containing 2% penicillin/streptomycin solution (v/v) (Gibco, Karlsruhe, Germany) and 1% amphotericin solution (v/v) (Biochrome, Berlin, Germany). Thereafter they were split open and the pulp was extirpated. Pulp explant cultures were propagated in DMEM (4.5g/l glucose, Sigma-Aldrich, Schnelldorf, Germany) supplemented with 1% (v/v) penicillin/streptomycin solution, 1mM ascorbic acid 2-phosphate (Sigma-Aldrich) and 10% (v/v) fetal calf serum (PAA, Cölbe Germany). For the herein specified experiments, pulp fibroblast cultures of passages 7 to 9 were used. All studies were conducted according to the Declaration of Helsinki Principles and were approved by the Local Ethics Commission/institutional review board (IRB) of the faculty of Medicine of the Johann Wolfgang-Goethe University (Frankfurt/Main, Germany). The Local Ethic Commission/IRB waived the need for consent.

### Biodentine^®^ suspensions

Biodentine^®^ (Septodont, Saint-Maur-des-fossés Cedex, France, powder: tricalcium silicate, dicalcium silicate, zirconium oxide, calcium carbonate; liquid: water, calcium chloride, polycarboxylate) was reconstituted according to the manufacturer’s instructions in its capsule in a cap mix (3M ESPE Neuss, Germany) and the paste was spread on a silicon molding tool to obtain Biodentine^®^ discs with a diameter of 5.1mm containing 0.14mg powder per disc. To obtain Biodentine^®^ suspensions, one, three or five Biodentine^®^ discs were incubated in 5ml of the cell-specific culture media (Table 1) containing 1% (v/v) fetal calf serum. The medium was collected and replaced every 24h. Media from 4 consecutive days were collected, centrifuged to obtain debris free suspensions and stored at -80°C.

**Table 1:** Biodentine^®^ concentrations of the Biodentine^®^ suspensions.

Biodentine^®^ discs per 5ml medium [number]	Biodentine^®^ powder per 5ml medium [mg]	Biodentine^®^ contact surface per ml culture medium [mm^2^]	Biodentine^®^ concentration in the suspension
1	0.14	14,58	low
3	0.42	43,74	moderate
5	0.70	72,90	high

Specifications of the applied Biodentine^®^ concentrations and the used synonyms in the manuscript.

### Influence of Biodentine^®^ on pulp fibroblasts

To evaluate the influence of the Biodentine^®^ suspensions, cell proliferation (BrdU incorporation, mitotic index, binucleotide index, Ki-67-rate), cell viability (MTT) and cell integrity (cell morphology and lactate dehydrogenase enzyme activity assay) were monitored. Pulp fibroblasts were statistically seeded at a cell density of 1x 10^5^ cells/ml in the above-specified DMEM if not otherwise stated in the analytical descriptions. After 24h, the culture medium was substituted with the Biodentine^®^ suspensions, fresh culture medium with 1% (v/v) fetal calf serum (control) or medium containing 1% (v/v) Triton X-100 (Merck, Darmstadt, Germany) and incubated for a further 24h.

### Morphological properties, mitotic- and binucleate index

Morphological properties as well as the mitotic- and binucleate index were monitored and analyzed after seeding pulp fibroblasts in a cell density of 1x 10^5^ cells/ml on glass cover slips in 24 well plates (greiner bio-one, Frickenhausen, Germany). After cell adherence the culture media were substituted with the above specified Biodentine^®^ suspensions or fresh culture medium as control. For binucleate induction cells were incubated with 1μg/ml cytochalasin B (Applichem, Darmstadt, Germany). Cell morphology was photodocumented every 2h for 24h with the incubator microscope unit IncuCyte (EssenBioScience, Hertfordshire, UK). After 24h cell were fixed with 4.5% formalin (Carl Roth, Karlsruhe, Germany) for 10min and washed with PBS^++^. Thereafter cell nuclei were counterstained with DAPI (Roche, Mannheim, Germany). Photographs were taken with a digital camera (Sony Cyber Shot 3.3, Sony, Cologne, Germany) connected to a Zeiss Axioskop (Zeiss, Oberkochen, Germany). The mitotic index and binucleate index were determined evaluating the number of cells showing mitotic phases (pro-metaphase to telophase) or binucleates (S1 Fig). Per condition and culture 1000 to 1089 cells were blindly analyzed.

### Flow cytometry

Pulp fibroblasts were seeded in a cell density of 5x10^5^ cell/ml. The medium was substituted on the following day as described above. After 24h cells were detached from the cell culture surface and fixed in 70% ethanol (Applichem) for at least 2h at -20°C. Thereafter the cells were washed with PBS containing 1% fetal calf serum. Cells were stained with either the monoclonal mouse anti-human Ki67, Alexa Fluor 647 conjugated (BD Biosciences Cat# 561126, RRID:AB_10611874) or the isotype control mouse IgG1, κ, Alexa Fluor 647 conjugated, Clone MOPC-21 (BD Biosciences Cat# 557714, RRID:AB_396823) according to the manufacturer’s instructions for 30min at room temperature. After washing twice with PBS containing 1% fetal calf serum to eliminate unbound antibodies, cell suspensions were incubated for 1h at 4°C with propidium iodide (Sigma-Aldrich). Cell suspensions were analyzed with an Accuri C6 flowcytometer (BD Biosciences, Heidelberg, Germany).

### Brom-2‘-desoxyuridin incorporation

The proliferative potential was analyzed with the Cell Proliferation ELISA BrdU (5-Brom-2‘-desoxyuridin, Roche, Mannheim, Germany) according to the manufacturer’s instructions. The test principal relies on the incorporation of BrdU during DNA replication as thymidine base analogue. Therefore the amount of BrdU incorporation can be used as analytical parameter for cell proliferation. The absorptions were measured with an ELISA-Reader (ASYS Expert 96, Biochrom, Cambridge, UK).

### Cell viability and cell integrity

Cell viability was evaluated using the MTT Cell Proliferation Assay (Trevigen, Gaithersburg, USA). The yellow tetrazolium salt is intracellularly reduced to purple formazan. After cell-lysis the amount of formazan can be measured. As analytical parameter for loss of cell integrity the cytosolic lactate dehydrogenase was chosen. The enzyme activity in cell free supernatants was measured with the Cytotoxicity Detection Assay (LDH, Roche). All assays were performed according to the manufacturers’ instructions and absorptions were measured with an ELISA-Reader.

### Collagen type I synthesis

To evaluate the effect of Biodentine^®^ on collagen type I synthesis, the concentration of the N-terminal domain of pro-collagen type I (P1NP) was quantified with the P1NP-Assay (P1NP-Elecsys assay, Roche Pharmaceuticals, Grenzach-Wyhlen, Germany) according to the manufacturer’s instructions as described [18]. Pulp fibroblasts were seeded at a cell density of 5x10^4^ cells/ml in 24-well plates. After 24h the culture medium was substituted either by fresh medium or by the above specified suspensions of Biodentine^®^. Additionally the high concentrated Biodentine^®^ suspension of day 1 was serially diluted (Table 2) before being added to the pulp fibroblast cultures. Cell-free supernatants were collected after 24h incubation with the respective media. P1NP was quantified.

**Table 2:** Serial dilution of the suspensions of five Biodentine^®^ discs of day 1.

Dilution factor	Biodentine^®^ suspension [ml]	Culture medium [ml]
1	3.0	0.0
1.25	2.8	0.7
1.75	2.0	1.5
2.5	1.4	2.1
3.5	1.0	2.5
5	0.7	2.8

Specifications of the serial dilution of the applied Biodentine^®^ suspensions of day 1.

### Enzyme-linked immunosorbent assay of TGF-β1

To determine the influence of Biodentine^®^ suspensions on TGF-β1 secretion, pulp fibroblasts were seeded and treated as described above. Cell-free supernatants were assayed using a commercial ELISA test kit (R&D, Wiesbaden-Nordenstadt, Germany) according to the manufacturer’s protocol.

### Immunhistochemical analysis of pulp fibroblasts

Pulp fibroblasts were seeded in a cell density of 5x 10^4^ cells/ml on glass cover slides. On the following day the cultures were treated with the Biodentine^®^ suspensions for 24h. Cultures destined for collagen type I staining were fixed in acetone for 10min at -20°C, cultures destined for TGF-β1 staining were fixed in 4% paraformaldehyd (Carl Roth) for 10min at room temperature. Thereafter the cultures were washed three times with PBS before blocking with 3% (v/v) bovine serum (Carl Roth) for 90min. After washing with PBS including 0.2% Tween (v/v) (Applichem) cells were stained overnight at 4°C with 1:500 diluted polyclonal rabbit anti-human collagen type 1 (Abcam Cat# ab34710, RRID:AB_731684) or 1:50 diluted monoclonal mouse anti-human TGF-β1 (Abcam Cat# ab27969, RRID:AB_778340). As secondary antibodies we used 1:500 diluted polyclonal goat anti-rabbit IgG (H+L), Alexa Fluor 546 conjugated (Thermo Fisher Scientific, Rockford USA, Cat# A-11010, RRID:AB_2534077) or polyclonal goat anti-mouse IgG (H+L), Alexa Fluor 546 conjugated (Thermo Fisher Scientific Cat# A-11003, RRID:AB_2534071) and incubated for 1h at room temperature. After washing the cells with 0.2% Tween PBS cell nuclei were counterstained with DAPI (Thermo Fisher Scientific) for 10min at room temperature and washed briefly in PBS and demineralised water. Coverslips were covered with Aqua Poly Mount (Polysciences, Hirschberg an der Bergstrasse, Germany) and fixed on microscope slides. Photographs were taken with a digital camera (Sony Cyber Shot 3.3, Sony, Cologne, Germany) connected to a Olympus BX-50 (Olympus, Hamburg, Germany). Fluorescence intensity of the collagen type I and TGF-β1 staining was quantified with Image J [19].

### Presentation of data and statistical analysis

All data are presented as mean values ± standard deviation of duplicates (Ki-67 analysis), triplicates (mitotic index, binucleate index), quadruplicate replicates (P1NP, TGF-β1) or sextuplicate replicates (BrdU, MTT, LDH). Statistical significance was evaluated by the Wilcoxon-Mann-Whitney U-test (BIAS, Frankfurt, Germany). Each set of data was related to the referring untreated control. Differences were considered significant at * p≤0.05; ** p≤0.01; *** p≤0.001. For significant differences between samples the magnitude of effect was calculated according to Rosenthal [20] (BIAS). E≥0.1 represents small effects, E≥0.3 represents moderate effects and E≥0.5 represents high effects (S1–S5 Tables).

## Results

Biodentine^®^ is used as a dentine substitute in restorative dental therapy and as a capping agent in dentistry [21–23]. To characterize the effects caused by this tricalcium silicate based cement, primary pulp fibroblasts were treated for 24h with different Biodentine^®^ suspensions. Elucidating concentration dependent effects (low, moderate and high Biodentine^®^ concentrations) and time-dependent effects (harvesting of the suspensions every 24h on 4 consecutive days) were achieved by incubation of pulp fibroblasts with the individual suspensions.

### Biodentine^®^ suspensions do not influence cell integrity or cell morphology of pulp fibroblasts

**Fig 1:**
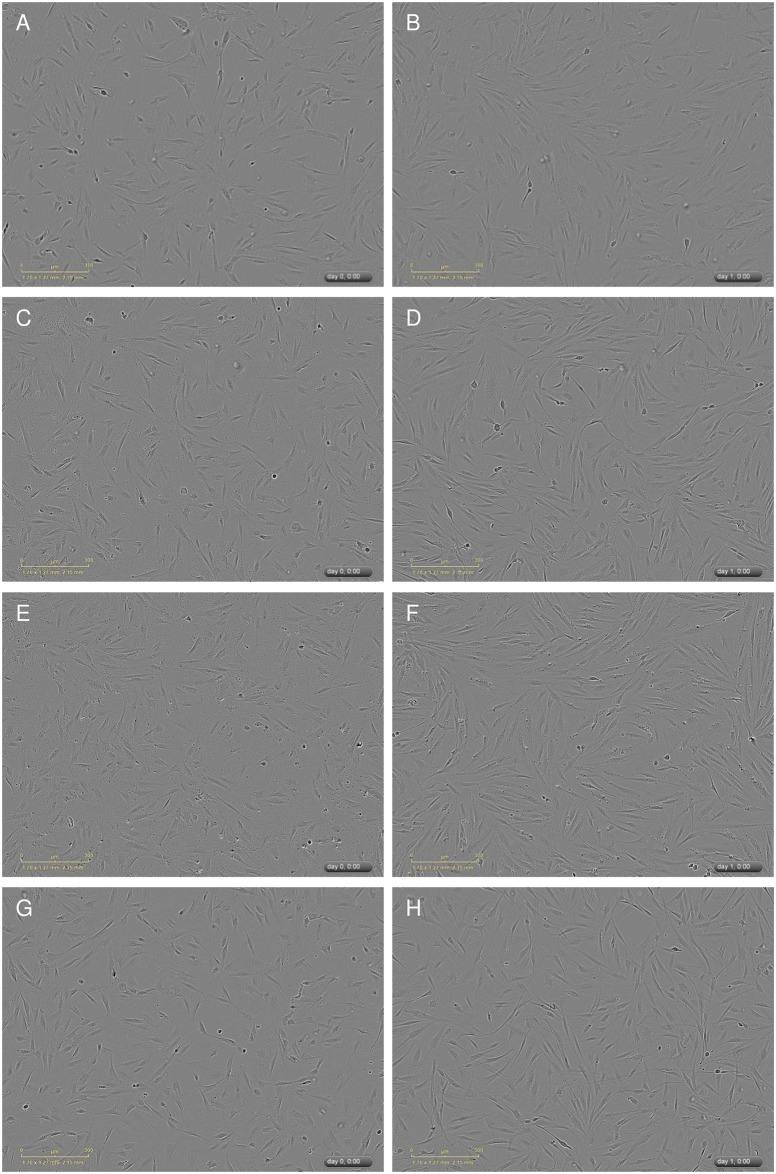
Cell morphology was not influenced by the used Biodentine^®^ suspensions. Cell morphology was monitored with an incubator microscope unit. Shown are exemplary pictures of the cell morphology immediately after application of the Biodentine^®^ suspensions or fresh control medium (A,C,E,G) and after 24h (B,D,F,H). Morphological changes could not be observed between the control (A,B) and the Biodentine^®^ suspensions of low (C,D), moderate (E,F) or high (G,H) concentration.

**Fig 2:**
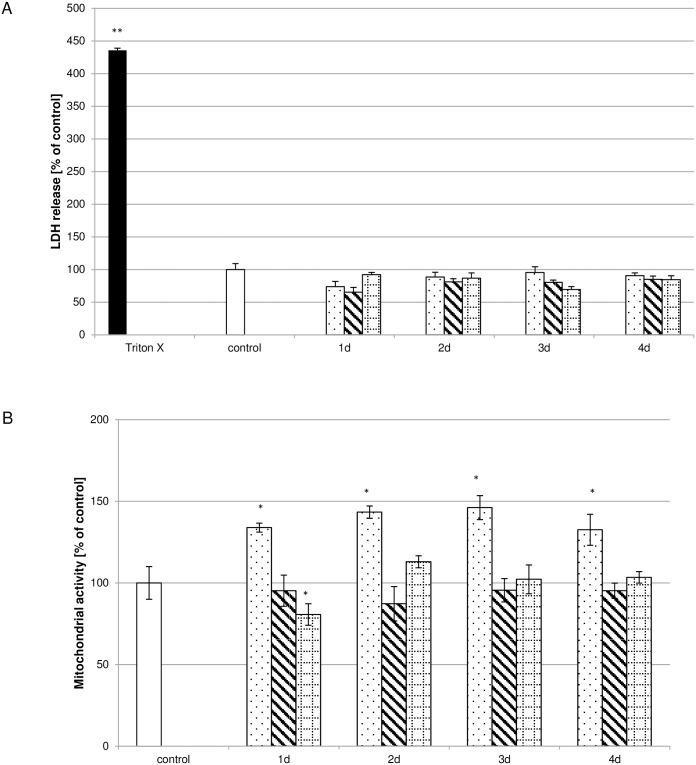
Biocompatibility of Biodentine^®^ suspensions. Cell integrity was monitored by assaying LDH activity (A; N = 324). No influence by the Biodentine^®^ suspensions of low (dotted bar), moderate (striped bar) or high (bricked bar) Biodentine^®^ concentrations on LDH activity could be observed in comparison to the control (white bar). LDH activity of the Triton X-100 (black bar) treated cultures was significantly increased. Biodentine^®^ suspensions containing low Biodentine^®^ concentrations increased cell viability whereas suspensions containing high Biodentine^®^ concentrations reduced cell viability (B; N = 270). The data displayed are representative of three independent experiments performed with comparable results. Average absorbance values (mean ± SD) from n = 6 replicates per experimental condition were calculated. * p ≤ 0.05; ** p≤0.01.

It could be shown that the morphology of the pulp fibroblasts treated with low (Fig 1C and 1D), moderate (Fig 1E and 1F) or high (Fig 1G and 1H) concentrated Biodentine^®^ suspensions did not differ from control cultures (Fig 1A and 1B) neither initially (Fig 1A, 1C, 1E and 1G) nor after 24h (Fig 1B, 1D, 1F and 1H). Furthermore it could be shown that the LDH activity (Fig 2A) in the cell free supernatants of cultures treated with suspensions of low (dotted bar), moderate (striped bar) or high (bricked bar) Biodentine^®^ concentrations was not increased or reduced in comparison to the control (white bar). As a positive control, pulp fibroblasts were treated with 1% Triton X-100 (black bar). The LDH activity of these samples was 200% higher than the control.

### Biodentine^®^ suspensions influence cell viability concentration and time dependently

Biodentine^®^ suspensions influence cell viability diversely. When incubating pulp fibroblasts with the suspensions containing low Biodentine^®^ concentrations, cell viability was increased by up to 46% of the control (Fig 2B). Monitoring cell viability after treatment with the suspensions containing moderate Biodentine^®^ concentrations showed no impact on pulp fibroblasts compared to the control whereas a distinct cell viability reduction could be observed in the case of treatment of pulp fibroblasts with the suspensions containing high Biodentine^®^ concentrations on day 1. The remaining suspensions containing high Biodentine^®^ concentrations had no impact on cell viability.

### Biodentine^®^ suspensions do not influence proliferation

**Fig 3:**
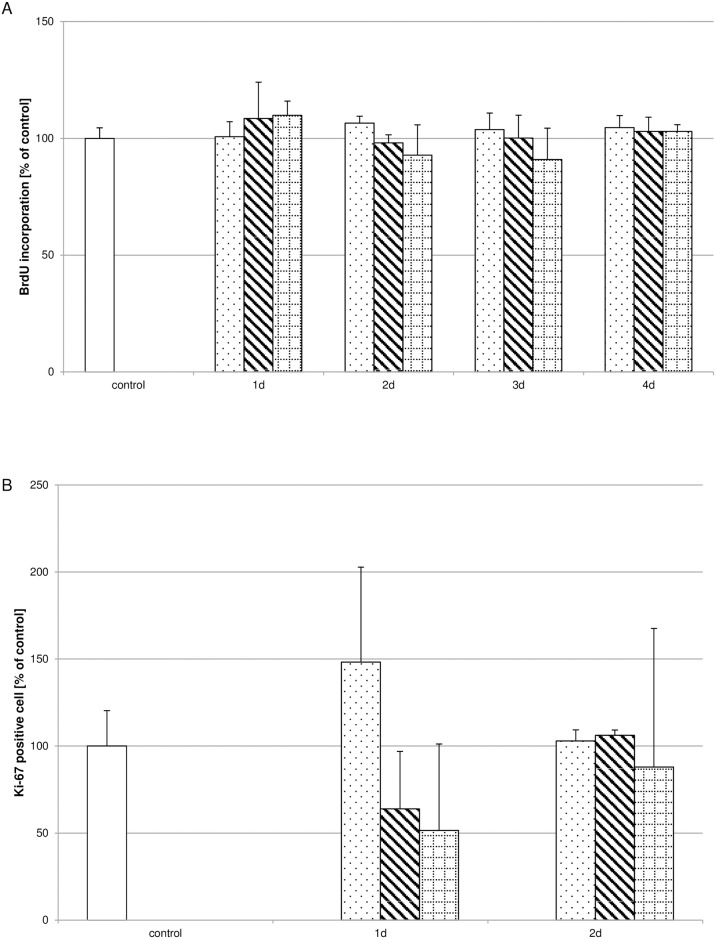
Influence of Biodentine^®^ suspensions on proliferative processes. Neither the incorporation of BrdU (A; N = 270) nor the number of Ki-67 positive cells (B) was significantly influenced by the Biodentine^®^ suspensions of low (dotted bar), moderate (striped bar) or high (bricked bar) Biodentine^®^ concentrations in comparison to the control (white bar). The data displayed are representative of three independent experiments (BrdU) or two independent experiments (Ki-67) performed with comparable results. Average absorbance values (mean ± SD) from n = 6 replicates per experimental condition calculated for the BrdU assay and the percental amount of Ki67-positive cells correlated to the control are shown.

**Fig 4:**
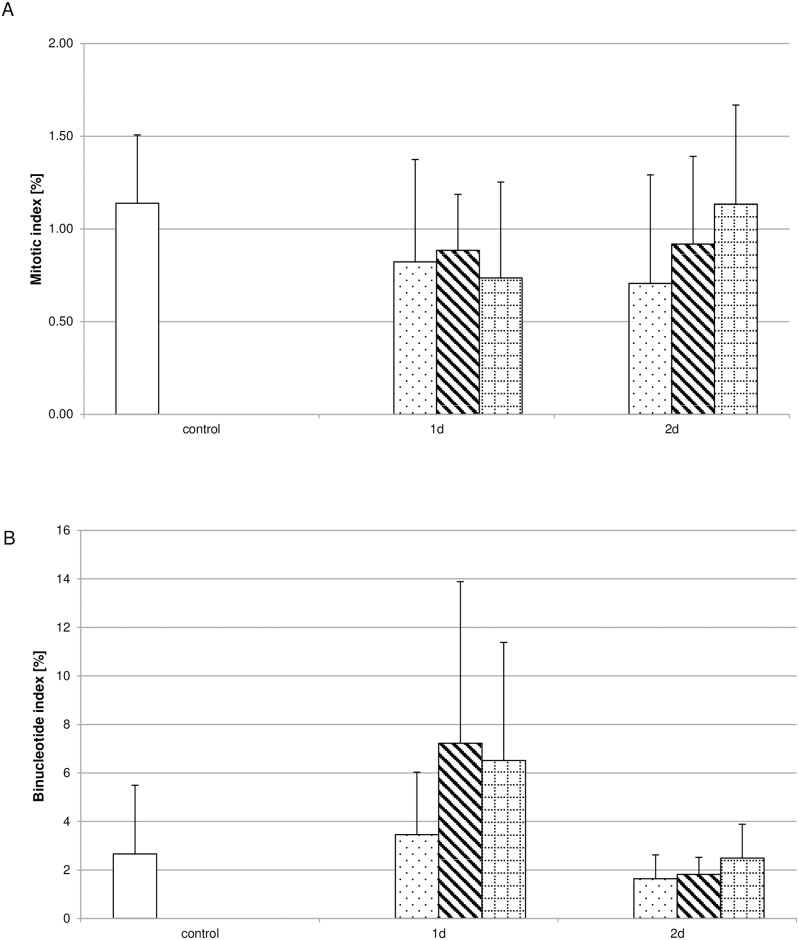
Biodentine^®^ suspensions did not influence the mitotic- or binucleotide index. The amount of cells showing characteristic mitotic phases (A) or binucleates (B) was correlated to the total cell number analyzed per treatment condition. No differences could be observed between the Biodentine^®^ suspensions of low (dotted bar), moderate (striped bar) or high (bricked bar) Biodentine^®^ or the controls (white bar). The data displayed are representative of three independent experiments performed with comparable results. For each culture condition approximately 1.000 cells were evaluated.

Analysing the influence of the different Biodentine^®^ suspensions concerning proliferation revealed that none of the suspensions used influenced the incorporation of BrdU during S-phase (Fig 3A) or the amount of Ki-67 positive cells (Fig 3B) significantly. To further validate this observation the mitotic index (Fig 4A) and the binucleotide index (Fig 4B) were determined. The number of cells showing characteristic mitotic phases (S1A–S1D Fig) were correlated to the total cell number for the mitotic index. For the binucleate index the cell number showing two nuclei (S1E and S1F Fig) after inhibiting cytokinesis by cytochalasin B were correlated to the total cell number. Neither index showed significant differences between the controls and the Biodentine^®^ suspension treated pulp fibroblast cultures.

### Biodentine^®^ suspensions decrease collagen type I synthesis and TGF-β1 secretion in pulp fibroblasts

**Fig 5:**
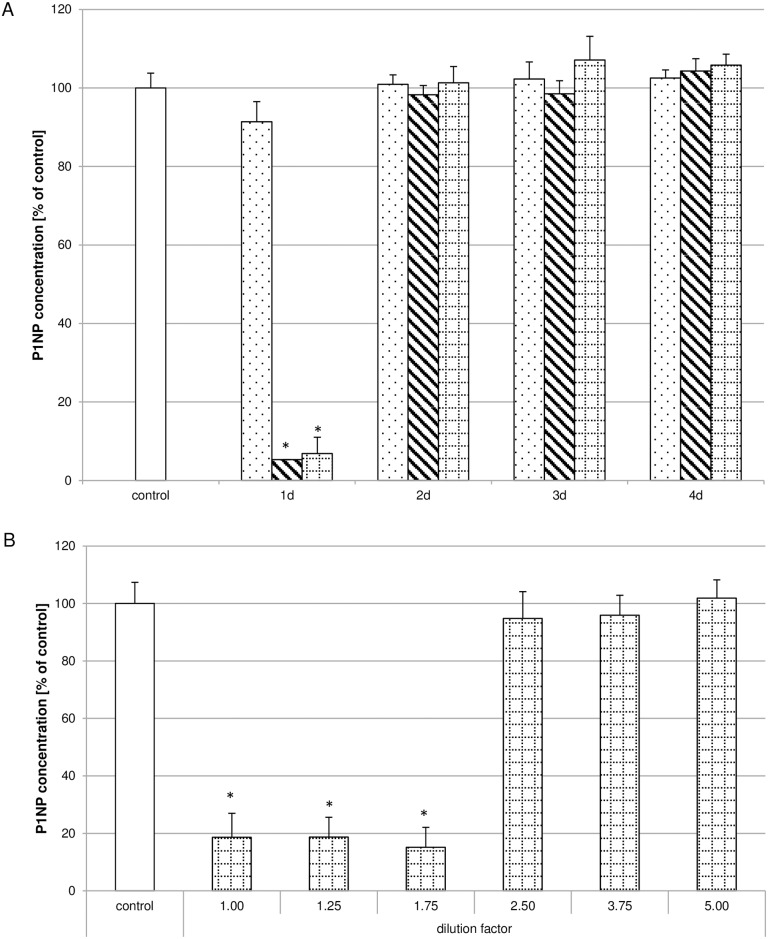
Biodentine^®^ suspensions decreased the concentration of the N-terminal domain of pro-collagen type I (P1NP). Collagen type I synthesis was evaluated measuring P1NP concentrations of cultures left untreated (white bar) or treated with Biodentine^®^ suspensions of low (dotted bar), moderate (striped bar) or high (bricked bar) Biodentine^®^ concentrations. P1NP in pulp cells treated with the Biodentine^®^ suspensions of day 1 was severely reduced (A; N = 180). Serial dilutions of the high Biodentine^®^ concentrations containing suspensions of day 1 revealed that up to a serial dilution of 1.75, P1NP secretion was reduced whereas dilutions from 2.5 did not influence P1NP secretion (B; N = 84). The data displayed are representative of three independent experiments performed with comparable results. Average absorbance values (mean ± SD) from n = 4 replicates per experimental condition were calculated. * p ≤ 0.05.

**Fig 6:**
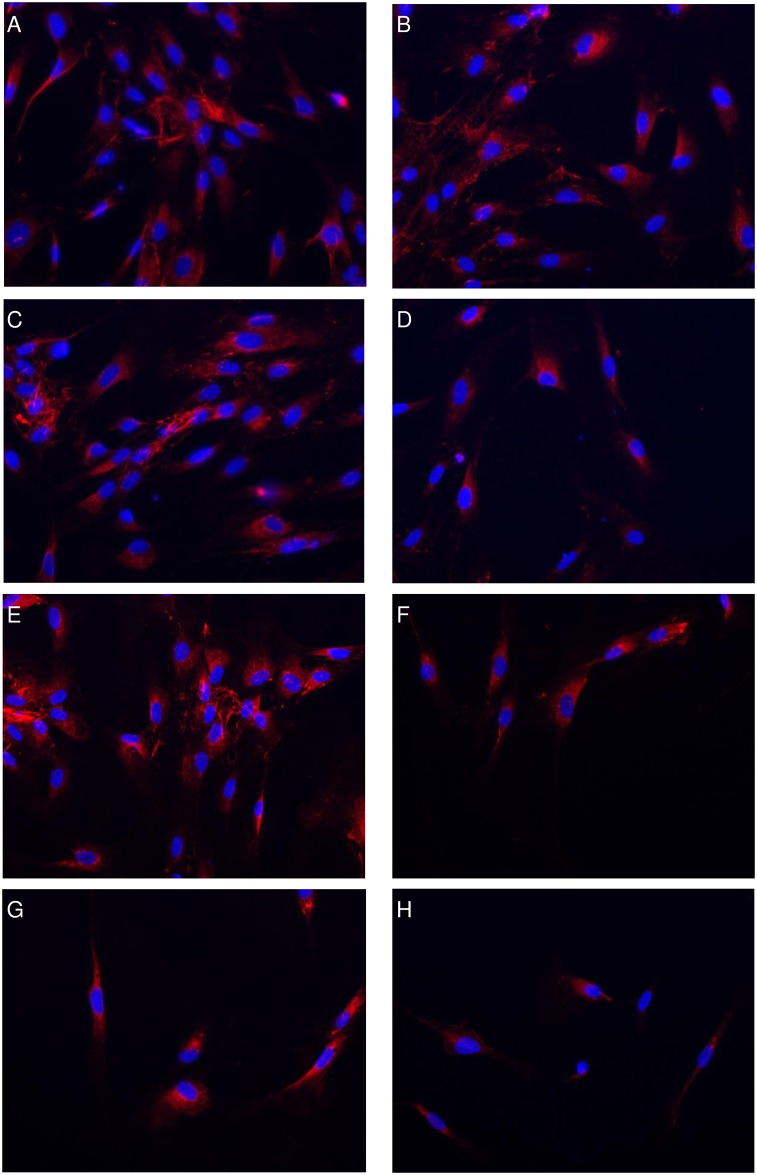
High concentrated Biodentine^®^ suspensions reduced collagen type I synthesis. The collagen type I synthesis of pulp fibroblasts treated with normal culture medium (A,B), Biodentine^®^ suspensions of low (C,D), moderate (E,F) or high (G,H) Biodentine^®^ concentrations of day 1 (A,C,E,G) or day 2 (B,D,F,H) were immunhistochemically monitored.

**Fig 7:**
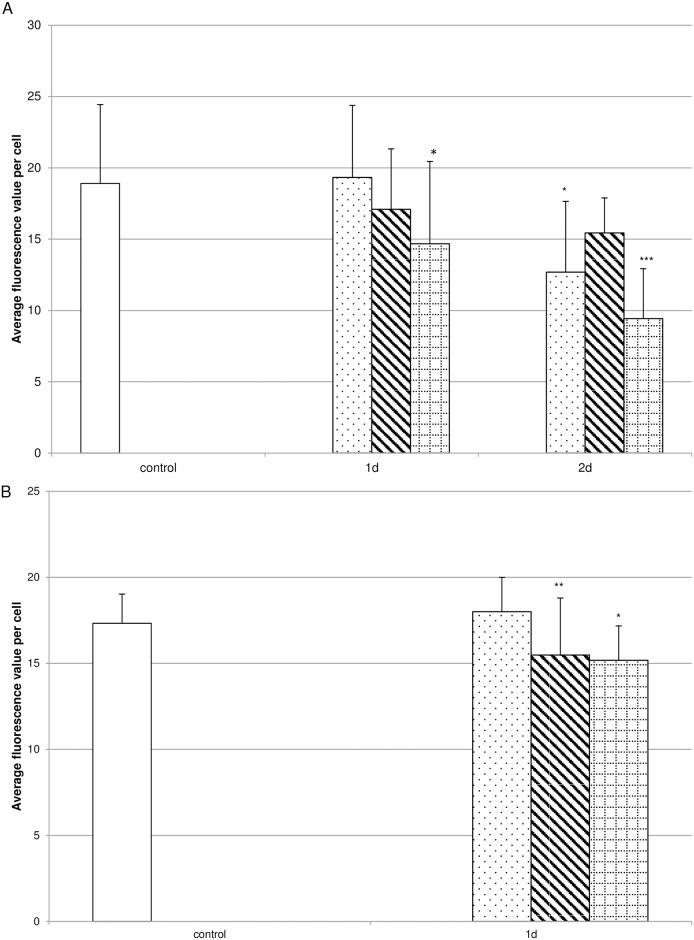
Biodentine^®^ suspensions decreased the collagen type I and TGF-β1 concentration. Collagen type I (A) and TGF-β1 (B) concentrations were measured by quantifying the fluorescence intensities of the respective immunhistochemical stainings of cultures treated with Biodentine^®^ suspensions of low (dotted bar), moderate (striped bar) or high (bricked bar) Biodentine^®^ concentrations in comparison to control cultures (white bar). The data displayed are representative of two independent experiments performed with comparable results. Average fluorescence intensity per cell (mean ± SD) from 14 to 17 individual cells per experimental condition were calculated. * p ≤ 0.05; *** p ≤ 0.001.

After revealing that cell viability was influenced by the Biodentine^®^ suspensions, the influence on collagen synthesis was investigated. Pulp fibroblasts were incubated with low, moderate or high Biodentine^®^ concentration containing suspensions. Whereas none of the suspension preparations containing low Biodentine^®^ concentrations influenced the concentration of P1NP and therefore collagen type I synthesis, the suspensions of day 1 of the preparations containing moderate or high Biodentine^®^ concentrations reduced collagen type I synthesis to 5% of the control (Fig 5A). The collagen type I synthesis reduction is limited to the suspension preparations of day 1. To verify these results the suspension preparations of day 1 containing high Biodentine^®^ concentrations were serially diluted before treating pulp fibroblasts. It could be shown that collagen type I synthesis was reduced up to a dilution factor of 1.75, but was comparable to the controls thereafter (Fig 5B). Immunhistochemical staining of collagen type I (Fig 6) revealed that no differences between the controls (Fig 6A and 6B) and the cultures incubated with suspensions containing low (C,D) to medium (E,F) Biodentine^®^ concentrations of day 1 and day 2 could be observed. After incubating pulp cells with high Biodentine^®^ concentrations a slightly more intense collagen type I staining could be observed in the region of the cell nucleus while the intensity in the cell periphery was lower than in all other cultures. Quantitative evaluation of the fluorescence intensity (Fig 7A) of the collagen type I staining revealed that the suspensions containing low Biodentine^®^ concentrations of day 2 and the suspensions containing high Biodentine^®^ concentrations of day 1 and day 2 showed significantly lower fluorescence intensities than the other cultures.

**Fig 8:**
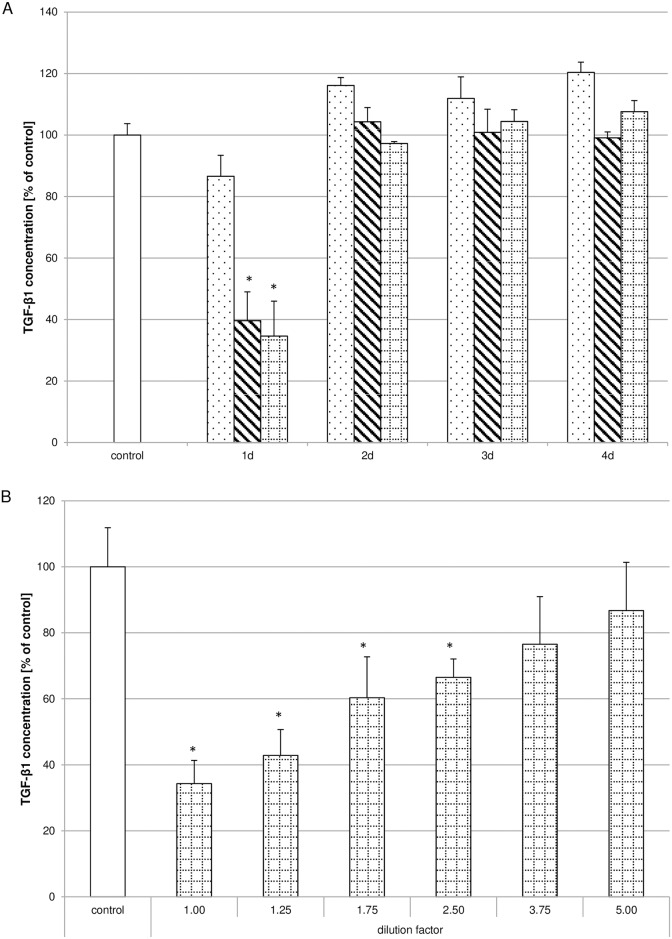
Biodentine^®^ suspensions decreased TGF-β1 secretion. TGF-β1 secretion was evaluated with a TGF-β1 ELISA. It could be shown that the Biodentine^®^ suspensions of low (dotted bar), moderate (striped bar) or high (bricked bar) Biodentine^®^ concentrations of day 1 reduced the TGF-β1 secretion in a concentration dependent way (A; N = 180) in comparison to the control (white bar). Serial dilutions of the high Biodentine^®^ concentrations containing suspensions of day 1 substantiated the concentration dependent TGF-β1 secretion inhibition (B; N = 84). The data displayed are representative of three independent experiments that were performed with comparable results. Average absorbance values (mean ± SD) from n = 4 replicates per experimental condition were calculated. * p ≤ 0.05.

Considering the importance of TGF-β1 for collagen synthesis as well as pulp regeneration, the influence of Biodentine^®^ suspensions on TGF-β1 secretion and distribution within the pulp fibroblasts (S2 Fig) was monitored. Pulp fibroblasts were treated according to the treatment regime for collagen type I synthesis. A clear concentration-dependent reduction of TGF-β1 concentration (Fig 7B) within the pulp fibroblasts could be observed after quantitative evaluation of the fluorescence intensity of the immunhistochemical stainings. Furthermore it could be shown that TGF-β1 secretion was reduced by incubation of the cells with the Biodentine^®^ preparations of day 1 (Fig 8A). Analysing TGF-β1 concentrations of the dilutions of the preparations of day 1 containing high Biodentine^®^ concentrations confirmed that Biodentine^®^ reduced TGF-β1 secretion concentration dependently (Fig 8B).

## Discussion

Exposition of pulp fibroblasts to Biodentine^®^ in our experimental setting revealed that none of the used suspensions influenced cell morphology or cell integrity according to the microscopic images and the quantification of the LDH activity. Although for similar components containing MTA, anti-proliferative [24, 25] as well as pro-proliferative effects [26] have been described, we could not determine any influence on proliferation. The inconsistent influence might be related to different cell species used or the different analytical parameters chosen for evaluating the proliferative potential of the analyzed dentine substitutes. In our study we did not determine the cell number or the time span to cover an artificially generated wound area but determined the amount of incorporated BrdU, the mitotic index as well as the development of binucleates after inhibiting cytokinesis. All of these aspects are dependent on cells performing mitosis. Coverage of an artificial wound is always associated to cell division but also to migratory processes. In vitro as well as in vivo Ki-67 is often used as a proliferative marker [27–30] because it is not expressed in G0 but in all other mitotic cell cycle phases. Our investigations showed that this parameter was also not significantly altered by Biodentine^®^ treatment. The viability of pulp cells increased when exposed to highly diluted Biodentine^®^ suspensions and decreased in a concentration-dependent manner. Similar effects have already been described for different cell species [26, 31–35] also considering time dependent effects [36]. Apart from biocompatibility, we were interested investigating the influence on the main component of the pulpal extracellular matrix. Collagen type I expression has been described to have a direct effect on hard dental tissue generation and mineralization [37, 38]. The influence of Biodentine^®^ and MTA on extracellular matrix proteins is versatile. It has been shown in a murine pulp cell line (OD21) [39] and human dental pulp cells [40] that calcium silicate containing materials decrease collagen type I mRNA expression [41, 42]. In our studies we could show that the described reduced collagen mRNA level correlates well with our collagen type I protein concentration analysis. Furthermore it has been described that Biodentine^®^ as wells as MTA induced collagen fibre degradation [43]. In contrast to our observations increased collagen synthesis [44–46] and faster formation of new dentine [23, 47, 48] and induction of osteogenesis [49] have been described for calcium silicate cements. A reason for the versatile observations might be that the concentrations of Biodentine^®^ and/or the surface area exposed to culture media is not consistent in the described studies. In our study e.g. we investigated surface contact areas per ml medium from 14,58mm^2^ to 70.92mm^2^ and therefore defined low Biodentine^®^ concentration as 14.58mm^2^ whereas Laurent et al. investigated contact surface areas of 0,05mm^2^ to 50mm^2^ [50]. Acknowledging the significance of TGF-β1 not only on collagen type I [51] synthesis but also on pulp regeneration in general [50, 52, 53], we could show that moderate to highly concentrated Biodentine^®^ suspensions reduced the initial TGF-β1 secretion. However, low Biodentine^®^ concentrations seem to induce TGF-β1 secretion as well as other growth factors [50, 54–56]. In our study serial dilutions of the highest Biodentine^®^ suspension substantiated the concentration-dependent effects on collagen type I synthesis and TGF-β1 secretion. We are therefore of the opinion that the TGF-β1 secretion decrease is responsible for the observed collagen type I synthesis reduction. Time and material specific differences of TGF-β secretion have been described showing that the secretion in the Portland cement group was higher than in the MTA treated group [47].

The present study is consistent with previous studies showing biocompatibility of Biodentine^®^. The clinical implication of the herein reported short term reduction of collagen type I synthesis has not been reported yet. Even though the influence of MTA and Biodentine^®^ on the generation of extracellular matrix proteins has been investigated in a variety of cell species involved in dentine regeneration contradictory influences showing stimulatory as wells as inhibitory effects have been reported. Clinical observations predominantly report induction of restorative tissue formation for tricalcium silicate cements. Due to the fact that Biodentine^®^ shows comparable physical and clinical qualities to MTA secondary criteria e.g. facilitated handling, applicability and faster setting time are taken into account.

Evaluation of the observed effects remains to be elucidated in comparison to other dentine substitutes as well as in other primary cell cultures and/or in in vitro three-dimensional tissue cultures

## Supporting Information

S1 Fig Exemplary pictures for identification of mitotic and binucleate cells. To determine the mitotic index after Biodentine^®^ suspension exposure cell nuclei showing characteristics of pro-metaphase to anaphase (A-D) were counted. The number of cells showing two cell nuclei after inhibiting cytokinesis by cytochalasin B (E, F) was used to define the binucleate index.
(TIF)

S2 Fig Exemplary pictures of TGF-β1 staining after incubation with Biodentine^®^ suspensions. TGF-β1 distribution in pulp fibroblasts treated with normal culture medium (A) Biodentine^®^ suspensions of low (B), moderate (C) or high (D) Biodentine^®^ concentrations of day 1 was immunhistochemically monitored.
(TIF)

S1 Table Detailed statistical information of the lactate dehydrogenase activity after Biodentine^®^ exposure. (DOCX)

S2 Table Detailed statistical information of cell viability analysis after Biodentine^®^ exposure. (DOCX)

S3 Table Detailed statistical information of the collagen type I synthesis after Biodentine^®^ exposure. (DOCX)

S4 Table Detailed statistical information of the collagen type I and TGF-β1 staining after Biodentine^®^ exposure. (DOCX)

S5 Table Detailed statistical information of the TGF-β1 secretion after Biodentine^®^ exposure. (DOCX)
