# Supplementary figures and images for: Influence of Biodentine® - A Dentine Substitute - On Collagen Type I Synthesis in Pulp Fibroblasts In Vitro
Source: PLoS One. 2016 Dec 9;11(12):e0167633. doi: 10.1371/journal.pone.0167633 (PMC5147936; doi:10.1371/journal.pone.0167633)

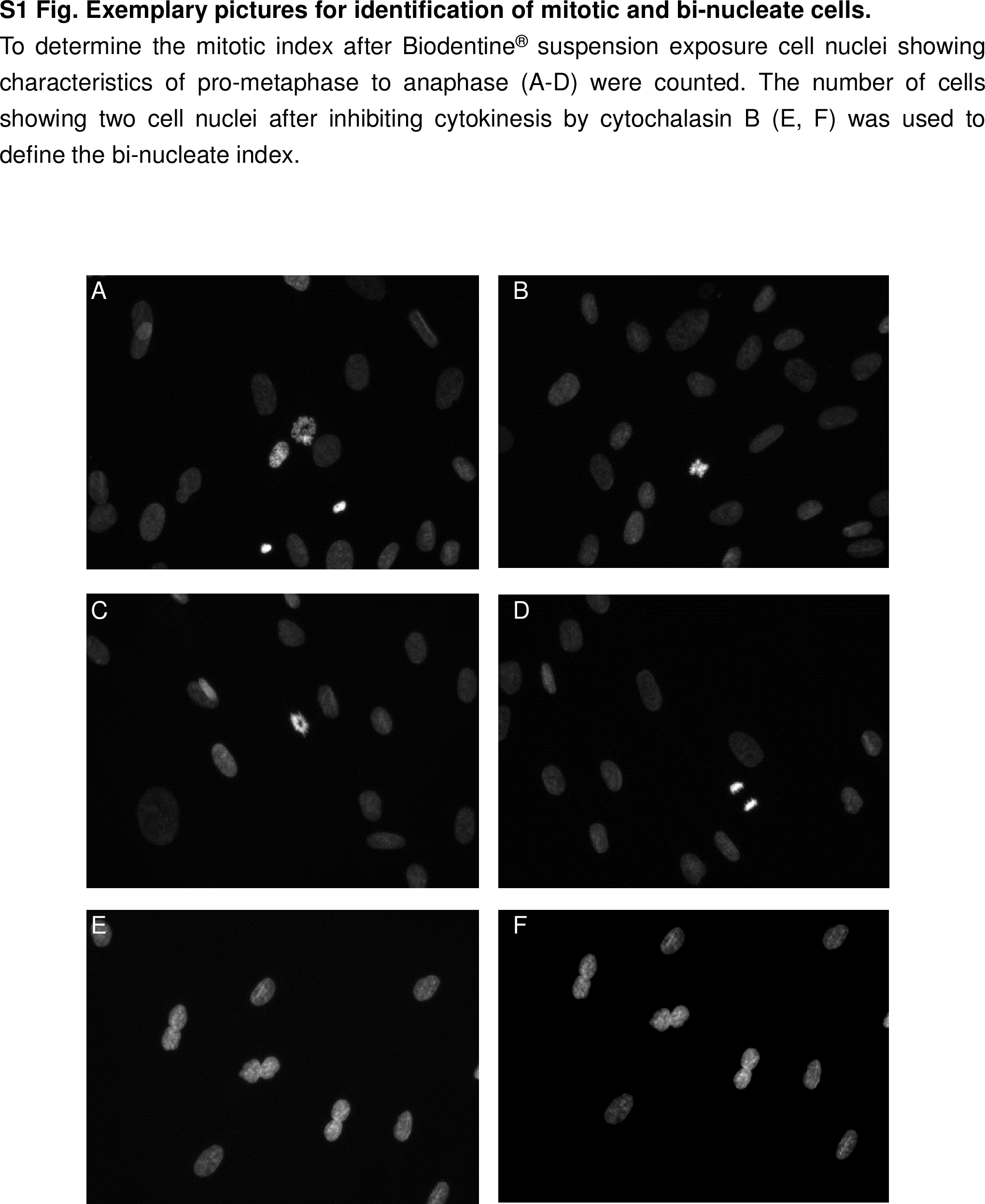

Supplement: S1 Fig — To determine the mitotic index after Biodentine® suspension exposure cell nuclei showing characteristics of pro-metaphase to anaphase (A-D) were counted. The number of cells showing two cell nuclei after inhibiting cytokinesis by cytochalasin B (E, F) was used to define the binucleate index. (TIF) [file pone.0167633.s001.tif]

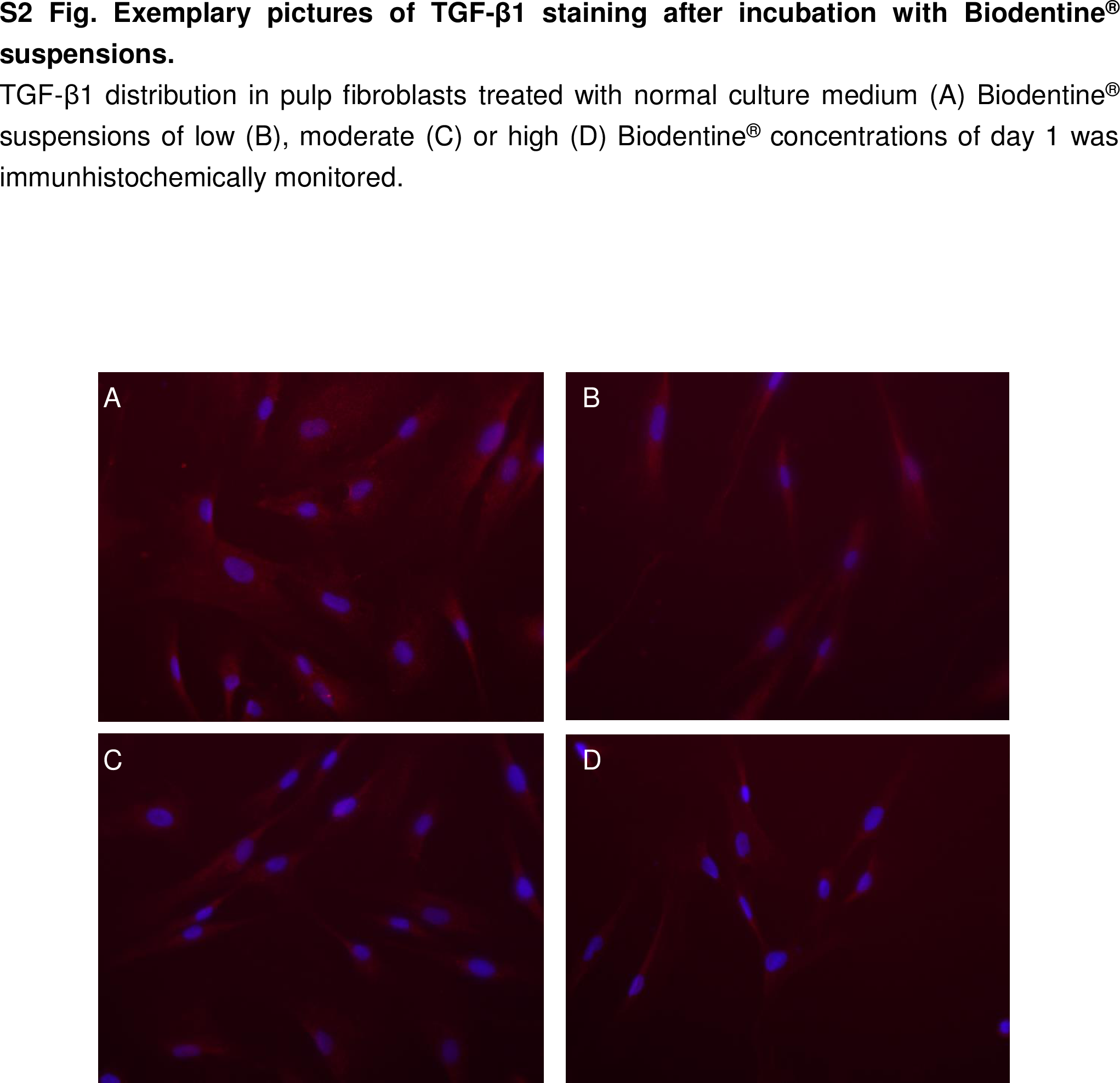

Supplement: S2 Fig — TGF-β1 distribution in pulp fibroblasts treated with normal culture medium (A) Biodentine® suspensions of low (B), moderate (C) or high (D) Biodentine® concentrations of day 1 was immunhistochemically monitored. (TIF) [file pone.0167633.s002.tif]
