# Supplementary material for: Influence of Biodentine® - A Dentine Substitute - On Collagen Type I Synthesis in Pulp Fibroblasts In Vitro
Source: PLoS One. 2016 Dec 9;11(12):e0167633. doi: 10.1371/journal.pone.0167633 (PMC5147936; doi:10.1371/journal.pone.0167633)
Supplement: S2 Table — (DOCX) [file pone.0167633.s004.docx]

**S2 Table. Detailed statistical information of cell viability analysis after Biodentine^®^ exposure.**

**MTT assay**

N= 270

low Biodentine^®^ concentration

| **against control** | **control** | **1d** | **2d** | **3d** | **4d** |
| --- | --- | --- | --- | --- | --- |
| **P value** | / | p=0.001265 | p=0.001265 | p=0.001265 | p=0.001265 |
| **E value** | / | 0.8439 | 0.8394 | 0.8439 | 0.8394 |
| **n per replicate** | 6 | 6 | 6 | 6 | 6 |
| **n in total** | 18 | 18 | 18 | 18 | 18 |

medium Biodentine^®^ concentration

| **against control** | **control** | **1d** | **2d** | **3d** | **4d** |
| --- | --- | --- | --- | --- | --- |
| **P value** | / | ns | ns | ns | ns |
| **E value** | / | ns | ns | ns | ns |
| **n per replicate** | 6 | 6 | 6 | 6 | 6 |
| **n in total** | 18 | 18 | 18 | 18 | 18 |

high Biodentine^®^ concentration

| **against control** | **control** | **1d** | **2d** | **3d** | **4d** |
| --- | --- | --- | --- | --- | --- |
| **P value** | / | p=0.001265 | ns | ns | ns |
| **E value** | / | 0.8364 | / | / | / |
| **n per replicate** | 6 | 6 | 6 | 6 | 6 |
| **n in total** | 18 | 18 | 18 | 18 | 18 |
