# Supplementary material for: Influence of Biodentine® - A Dentine Substitute - On Collagen Type I Synthesis in Pulp Fibroblasts In Vitro
Source: PLoS One. 2016 Dec 9;11(12):e0167633. doi: 10.1371/journal.pone.0167633 (PMC5147936; doi:10.1371/journal.pone.0167633)
Supplement: S4 Table — (DOCX) [file pone.0167633.s006.docx]

**S4 Table. Detailed statistical information of the collagen type I and TGF-β1 staining after Biodentine^®^ exposure.**

**Collagen type I staining**

N=95

low Biodentine^®^ concentration

| **against control** | **control** | **1d** | **2d** |
| --- | --- | --- | --- |
| **P value** | / | ns | 0,019448 |
| **E value** | / | / | 0,3676 |
| **n in total** | 16 | 14 | 13 |

medium Biodentine^®^ concentration

| **against control** | **control** | **1d** | **2d** |
| --- | --- | --- | --- |
| **P value** | / | ns | ns |
| **E value** | / | / | / |
| **n in total** | 16 | 13 | 10 |

high Biodentine^®^ concentration

| **against control** | **control** | **1d** | **2d** |
| --- | --- | --- | --- |
| **P value** | / | 0.032651 | 0,000003 |
| **E value** | / | 0.3257 | 0,6698 |
| **n in total** | 16 | 15 | 14 |

**TGF-β1 staining**

N=76

low Biodentine^®^ concentration

| **against control** | **control** | **1d** |
| --- | --- | --- |
| **P value** | / | ns |
| **E value** | / | / |
| **n in total** | 13 | 18 |

medium Biodentine^®^ concentration

| **against control** | **control** | **1d** |
| --- | --- | --- |
| **P value** | / | 0,009721 |
| **E value** | / | 0,4041 |
| **n in total** | 13 | 27 |

high Biodentine^®^ concentration

| **against control** | **control** | **1d** |
| --- | --- | --- |
| **P value** | / | 0.015591 |
| **E value** | / | 0.4350 |
| **n in total** | 13 | 18 |
