# Supplementary material for: Influence of Biodentine® - A Dentine Substitute - On Collagen Type I Synthesis in Pulp Fibroblasts In Vitro
Source: PLoS One. 2016 Dec 9;11(12):e0167633. doi: 10.1371/journal.pone.0167633 (PMC5147936; doi:10.1371/journal.pone.0167633)
Supplement: S5 Table — (DOCX) [file pone.0167633.s007.docx]

**S5 Table. Detailed statistical information of the TGF-β1 secretion after Biodentine^®^ exposure.**

**TGF-β1 ELISA**

N= 180

low Biodentine^®^ concentration

| **against control** | **control** | **1d** | **2d** | **3d** | **4d** |
| --- | --- | --- | --- | --- | --- |
| **P value** | / | ns | ns | ns | ns |
| **E value** | / | / | / | / | / |
| **n per replicate** | 4 | 4 | 4 | 4 | 4 |
| **n in total** | 12 | 12 | 12 | 12 | 12 |

medium Biodentine^®^ concentration

| **against control** | **control** | **1d** | **2d** | **3d** | **4d** |
| --- | --- | --- | --- | --- | --- |
| **P value** | / | p=0.0285715 | ns | ns | ns |
| **E value** | / | 0.8214 | / | / | / |
| **n per replicate** | 4 | 4 | 4 | 4 | 4 |
| **n in total** | 12 | 12 | 12 | 12 | 12 |

high Biodentine^®^ concentration

| **against control** | **control** | **1d** | **2d** | **3d** | **4d** |
| --- | --- | --- | --- | --- | --- |
| **P value** | / | p=0.028571 | ns | ns | ns |
| **E value** | / | 0.8264 | / | / | / |
| **n per replicate** | 4 | 4 | 4 | 4 | 4 |
| **n in total** | 12 | 12 | 12 | 12 | 12 |

Dilution

N=84

| **against control** | **control** | **1.00** | **1.25** | **1.75** | **2.5** | **3.75** | **5.00** |
| --- | --- | --- | --- | --- | --- | --- | --- |
| **P value** | / | p=0.028571 | p=0.028571 | p=0.028571 | p=0.028571 | ns | ns |
| **E value** | / | 0.8214 | 0.8214 | 0.8165 | 0.8165 | / | / |
| **n per replicate** | 4 | 4 | 4 | 4 | 4 | 4 | 4 |
| **n in total** | 12 | 12 | 12 | 12 | 12 | 12 | 12 |
